# Diffuse Idiopathic Pulmonary Neuroendocrine Cell Hyperplasia With Progression to Neuroendocrine Tumor

**DOI:** 10.7759/cureus.13297

**Published:** 2021-02-12

**Authors:** Débora Sousa, Filipa Rocha, Bernardo Baptista, Alexandra Bayão Horta

**Affiliations:** 1 Internal Medicine Department, Hospital da Luz Lisboa, Lisbon, PRT

**Keywords:** pulmonary neuroendocrine tumors, dipnech, diffuse idiopathic pulmonary neuroendocrine cell hyperplasia, carcinoid tumor, preneoplastic lesions

## Abstract

Diffuse idiopathic pulmonary neuroendocrine cell hyperplasia (DIPNECH) is a parenchymal lung disease characterized by a proliferation of neuroendocrine cells in the bronchial wall, with possible local invasion and occasional development of tumorlets. It is considered to be a precursor lesion as it can progress to neuroendocrine tumors (NETs). At presentation, approximately one-half of patients with DIPNECH have a synchronous diagnosis of NET.

Here, we present the case of a 95-year-old woman with progressive exertional dyspnea. She was found to have an obstructive airway syndrome and long-lasting progressive bilateral pulmonary nodules, with a distribution and growth pattern suggestive of DIPNECH, as well as possible progression to NET in the larger lesions. A transthoracic needle aspiration biopsy of a pulmonary nodule was performed, confirming the diagnosis of NET, evolving from DIPNECH.

## Introduction

Diffuse idiopathic pulmonary neuroendocrine cell hyperplasia (DIPNECH) defined by diffuse proliferation of neuroendocrine cells on the bronchial wall is a rare condition, and its true prevalence is unknown. It is a primary entity and must be distinguished from proliferation of secondary neuroendocrine cells, reactive to other pathological processes [1-3]. Its first descriptions came from incidental findings on biopsies of lung nodules, and, consequently, it was initially described as a histological entity and recognized by the World Health Organization as a premalignant disorder [1,3]. Based on accumulating evidence from case reports, DIPNECH is now considered a syndrome of unknown cause, with typical clinical, radiological, and histopathological findings [3,4].

Clinically, it has been predominantly observed in middle aged (40 to 60-years old) nonsmoking females who present with an insidious onset of exertional dyspnea, cough, or wheezing [4]. As pulmonary function tests frequently show a pure or mixed obstructive pattern, the clinical picture is usually misinterpreted as being caused by asthma or chronic obstructive pulmonary disease [2-4].

Typical computer tomography (CT) findings include diffuse round-to-ovoid lung nodules of solid density scattered in the parenchyma. More than 60% of DIPNECH patients have multiples nodules with one or more dominant lesions, corresponding to carcinoid tumors. Bronchial wall thickening and bronchiectasis can also be seen [2,3].

Neuroendocrine cell hyperplasia (NECH) can be superficial and isolated or invade the basement membrane and form tumorlets (cell aggregates <5 mm) or carcinoid tumors (if >5 mm) [1,2,4]. Histologically, DIPNECH is characterized by the presence of a variable number of tumorlets and/or carcinoid tumors [1,4]. Neuroendocrine origin is confirmed by cell expression of cluster of differentiation (CD)56, synaptophysin, and chromogranin A [3]. DIPNECH is strongly associated with peribronchiolar fibrosis and constrictive obliterative bronchiolitis due to chronic inflammatory cell infiltrates, wall thickening, and fibrosis, thought to be mediated by neuroendocrine cell products [2]. Interestingly, different expression of thyroid transcription factor 1 and CD10, high levels of somatostatin receptors, and the activation of mammalian target of rapamycin (mTOR) pathway has been shown in patients with DIPNECH syndrome [4].

The clinical course can range from long-term stability, slowly progressive functional decline or rapid clinical decline in high-grade tumors, or severe bronchiolitis with respiratory failure [3,4]. Despite being considered a premalignant condition and the identification of a synchronous carcinoid tumor in one-half of patients at DIPNECH diagnosis, the rate and risk factors for progression to neuroendocrine tumor (NET) are unknown [3].

## Case presentation

A 95-year-old woman was admitted due to a six-week history of progressive exertional dyspnea. She also complained of orthopnea and lower extremity edema. Fever, cough, chest pain, or other symptoms suggestive of carcinoid syndrome were absent. She had a history of hypertension, type 2 diabetes, and asthma, which was diagnosed at the age of 70 (25 years ago). She was medicated with metformin 1000 mg twice a day, furosemide 20 mg a day, acetylsalicylic acid 100 mg a day, and olmesartan 20 mg a day. She was a nonsmoker and had no history of occupational or environmental exposure to toxic fumes.

On physical examination, she was tachypneic and had a peripheral oxygen saturation of 93% with supplemental oxygen at 1 L/minute delivered by a nasal canula. She had normal breath sounds on auscultation and had lower extremities pitting edema, which extended symmetrically into both knees. There was no jugular venous distention.

Admission blood tests depicted a hemoglobin of 12.8 g/dL, a normal white blood cell count, and a C-reactive protein of 1 mg/dL. She had normal kidney function, normal serum troponin I, N-terminal pro b-type natriuretic peptide (NT-proBNP) of 100 pg/mL, and D-dimer of 1.28 ug/mL. Initial arterial blood gas analysis revealed hypoxemia and signs of chronic respiratory failure, with a pH of 7.39, pCO_2_ 69 mmHg, pO_2_ 52 mmHg, and HCO_3_ of 41.8 mEq/L.

**Figure 1:**
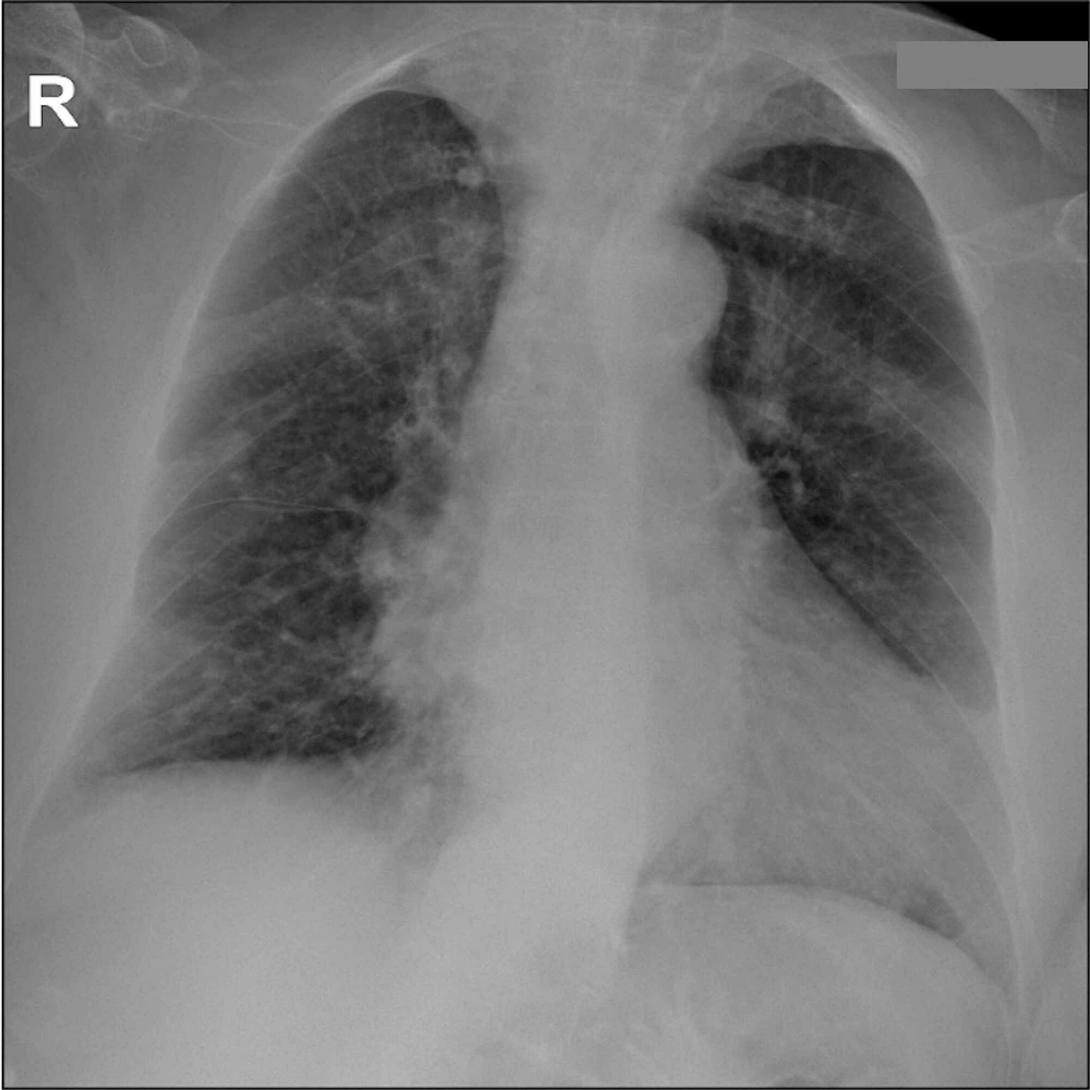
Chest radiograph showing a bilateral reticular pattern.

**Figure 2:**
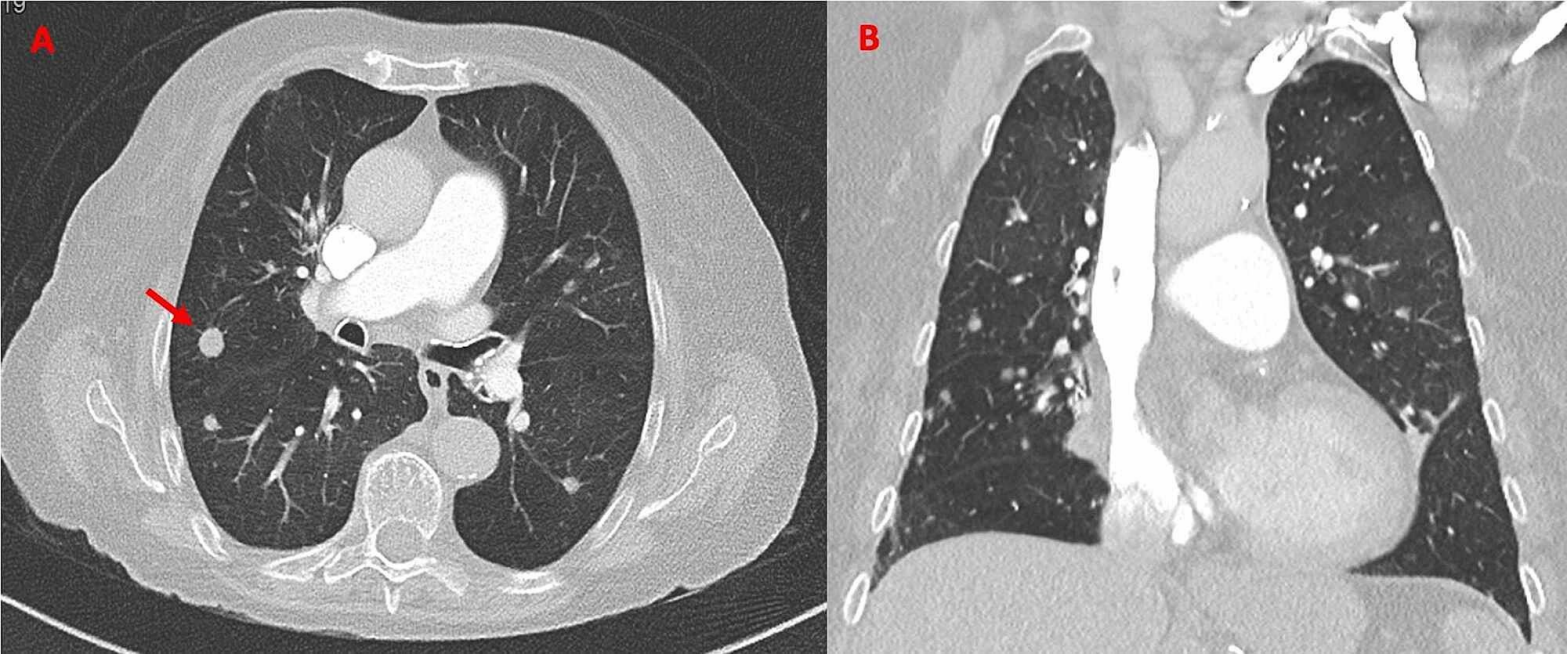
Chest CT angiography from 2019 (admission) showing nodules in the lung parenchyma, with the largest located in the right superior lobe, with the biggest diameter of 9 mm (arrow). Panel A: transversal plane; Panel B: coronal plane. CT, computed tomography

An initial chest radiograph showed a bilateral reticular pattern (Figure 1), and a chest CT angiography excluded pulmonary embolism and showed bilateral nodules with random distribution, the biggest one 9 mm in diameter (Figure 2), bronchial wall thickening, and bronchiectasis, as well as hilar and mediastinal adenopathies.

**Figure 3:**
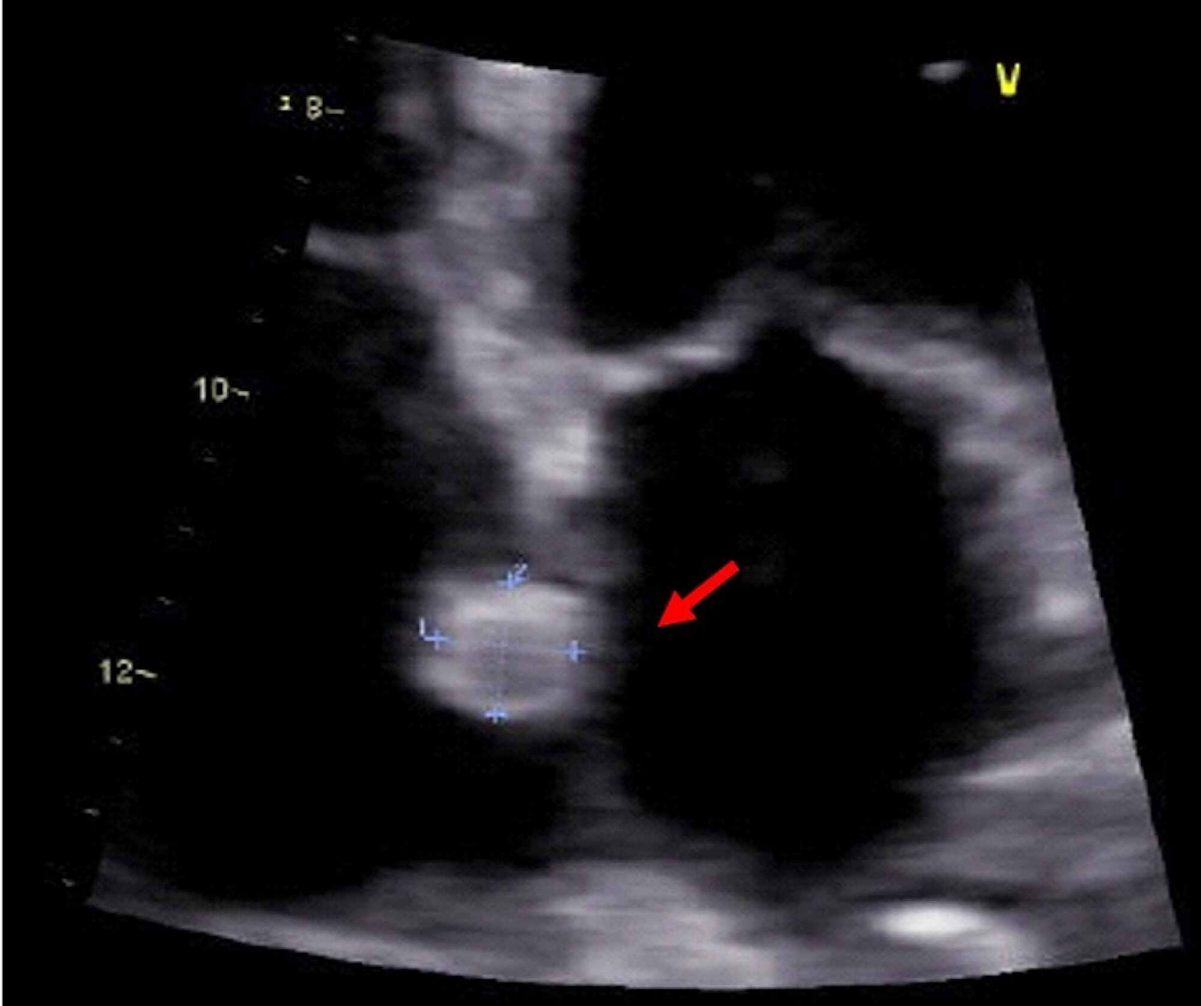
Echocardiogram showing a nodular image adherent to the right atria (arrow).

Transthoracic echocardiogram showed a normal left ventricle ejection fraction, with no evidence of left heart disease and no visible left-right shunt, but revealed a tricuspid regurgitation with moderate pulmonary hypertension (PH) (estimated pulmonary artery systolic pressure [PASP] of 53,57 mmHg) and described a well-defined homogeneous nodular image adherent to the right atria (Figure 3). These were new findings as our patient had an echocardiogram from three years earlier in which the estimated PASP was 25 mmHg with no apparent nodular image in the right atria.

**Figure 4:**
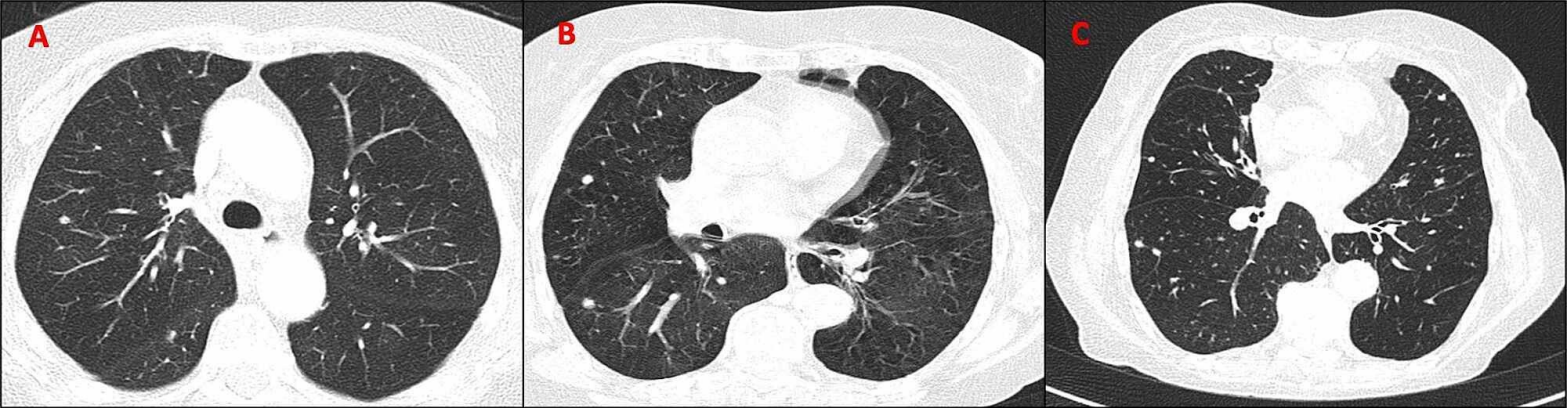
Chest CTs from 2008 (Panel A), 2011 (Panel B), and 2018 (Panel C) showing the progression in size and distribution of a parenchymal micronodular pattern. CT, computed tomography

A review of her previous examinations showed that the bilateral parenchymal micronodular pattern was already present in a 2008 CT scan (11 years before she was admitted to our hospital) and progressed in size and distribution in the follow-up examinations (Figure 4). Previous lung function tests were compatible with a reversible peripheral obstructive airway syndrome, with hyperinflation and normal carbon monoxide diffusion.

In summary, our patient had long-lasting progressive bilateral pulmonary nodules and an obstructive airway syndrome, with signs of chronic respiratory failure, acutely exacerbated. CT scans were assessed by a senior radiologist who suggested, based on the distribution, size, and growth of the nodules, a diagnosis of DIPNECH, with a possible progression to NET in the larger lesions. At this point, we also measured chromogranin A in the blood, which was 4 nmol/L (normal value <3 nmol/L).

**Figure 5:**
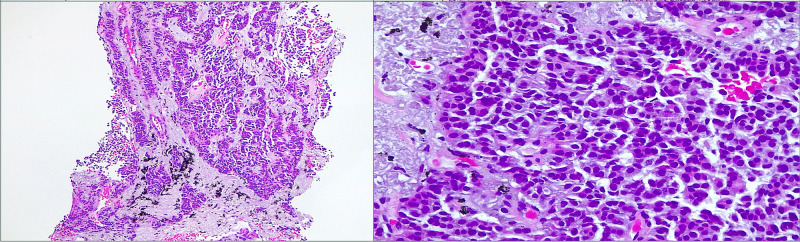
Pathological examination: microscopic examination.

**Figure 6:**
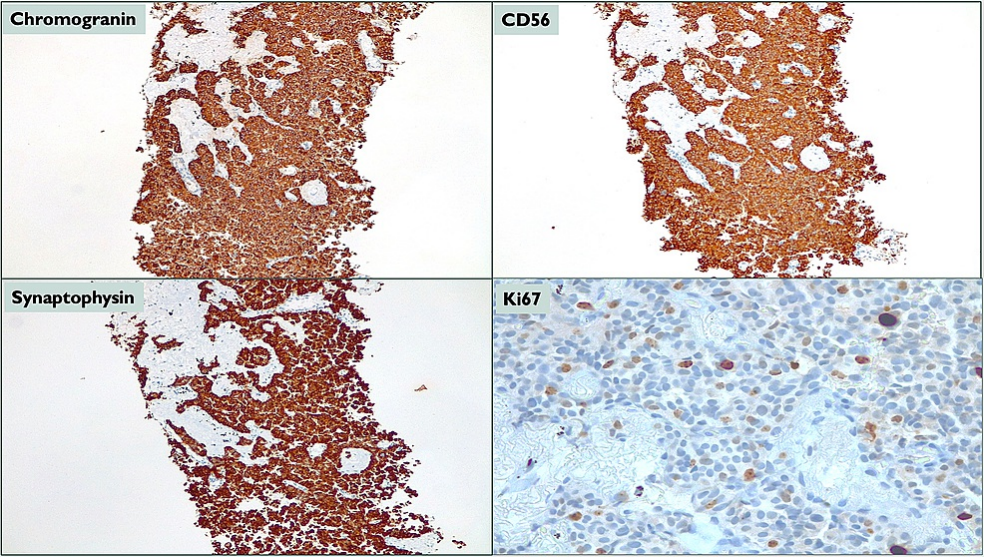
Pathological examination: immunohistochemical features.

At this stage, a transthoracic needle aspiration biopsy of the biggest pulmonary nodule was performed, and pathological examination showed small hyperchromatic cells, without mitosis or necrosis (Figure 5), strongly positive for CD56, synaptophysin, and chromogranin A (Figure 6) and a Ki-67 index between 1% and 3%, consistent with a typical carcinoid tumor (well-differentiated NET, low grade). We were then able to establish the diagnosis of a pulmonary NET, evolving from DIPNECH.

To characterize the right atria nodule, we proposed a transesophageal echocardiogram as well as a gallium 68 DOTATOC positron emission tomography (PET) scan to confirm if the heart nodule corresponded to metastatic disease. Due to the patient’s advanced age, a conservative approach was chosen, and both examinations were dismissed.

Initial differential diagnosis included all causes of pulmonary nodules, including benign, malignant, infectious, and inflammatory causes. Based on the long course of disease and the chronological evaluation of the CTs that our patient had undergone over the years, we were able to narrow the spectrum of possibilities as both were compatible with the DIPNECH syndrome. Definitive diagnosis was obtained with the biopsy results.

The patient was treated with a corticosteroid inhaler, a long-acting β-agonist inhaler, and diuretics, with transitory improvement of the symptoms. Following a multidisciplinary team meeting involving Internal Medicine, Oncology, Pulmonology, and Radiology departments, we decided to adopt a conservative approach and not to start any directed treatment, such as somatostatin analogs.

The patient was discharged from the hospital after one week. At six-month follow-up, she maintained symptoms of right heart failure and there was a decline in performance status. She died eight months after the diagnosis.

## Discussion

As DIPNECH is a rare condition, available literature comes from small retrospective studies and case report series [5]. Additionally, the term DIPNECH has been indiscriminately used to describe two different subsets of patients: those with purely histological findings and those with the clinical, functional, and radiological syndrome, as described in our patient. Consequently, data on this entity are limited, and validated diagnostic criteria and recommendations on workup and management are still to be defined [4].

Most published cases report DIPNECH as a diagnosis based solely on a histological incidental finding and only a few have been suspected on clinical grounds [3,5]. We present the case of a 95-year-old nonsmoker female who met the clinical, functional, radiological, and histological proposed features of DIPNECH syndrome. She presented 25 years earlier with a reversible obstructive airway syndrome misdiagnosed as late asthma, and later pulmonary micronodules were found. Serial CT scans during an 11-year period showed growth of the nodular pattern which led to the clinical suspicion of DIPNECH, and a tissue biopsy confirmed neuroendocrine cell proliferation consistent with a typical carcinoid tumor. Although the sample was insufficient to evaluate the bronchial mucosa, the presence of bronchiectasis and long-standing peripheral obstructive airway syndrome with hyperinflation and normal carbon monoxide diffusion led us to the assumption that she might have had constrictive bronchiolitis. This would explain the global respiratory insufficiency and PH. Another contributor to the PH may have been the progression to NET, as her chromogranin A levels were elevated, and increased vasoactive substances produced by neuroendocrine cells can increase pulmonary artery pressure [6].

NETs are still considered infrequent neoplasms and 90% arise from the gastrointestinal (GI) tract [7,8]. Lung NETs are the second most common location and metastization usually occurs to the mediastinal lymph nodes, liver, and bone [9]. Carcinoid heart disease classically involves the right heart and is caused by the increased secretion of vasoactive substances, such as serotonin, in the context of carcinoid syndrome [8,9]. Cardiac metastases are rarely reported in NETs, and most refer to primary GI tumors [7,9]. They are described as homogenous, well-circumscribed, noninfiltrative intramyocardial masses on the atria or ventricle, which can embolize or cause obstructive symptoms [9]. Our patient’s echocardiogram showed a nodular image adherent to the right atria, with characteristics compatible with a cardiac metastasis. Although further investigation was not possible and a thrombotic etiology could not be excluded, we speculate that it could correspond to a cardiac metastasis of her lung NET. Considering lung lymphatic drainage and the presence of hilar and mediastinal adenopathies, metastization to the right atria seems plausible. A biopsy or excision or alternatively a PET DOTATOC would have been helpful in identifying the possible neuroendocrine origin [7,9].

Management decisions depend on patient’s life expectancy, clinical status, and personal preferences. In patients with mild and stable presentation, observation alone may suffice [1,3]. Therapeutic options may include oral and inhaled steroids, chemotherapy, surgical lung resection, and lung transplantation. Demonstration of activation of the mTOR pathway or somatostatin receptor expression can represent additional therapeutic targets [3].

## Conclusions

DIPNECH is an extremely rare condition that can take a long time to diagnose. Although it is being recognized with increasing frequency, the evidence regarding its diagnosis and management is scarce.

The diagnosis of DIPNECH requires a high index of suspicion. We report a case of a patient with long-standing DIPNECH with progression to NET diagnosed at the age of 95, with probable nodal and cardiac metastization, in hopes of contributing to a better understanding of this rare disorder.
